# Analysis of *TNFAIP3*, a feedback inhibitor of nuclear factor-κB and the neighbor intergenic 6q23 region in rheumatoid arthritis susceptibility

**DOI:** 10.1186/ar2650

**Published:** 2009-03-17

**Authors:** Rebeca Dieguez-Gonzalez, Manuel Calaza, Eva Perez-Pampin, Alejandro Balsa, Francisco J Blanco, Juan D Cañete, Rafael Caliz, Luis Carreño, Arturo R de la Serna, Benjamin Fernandez-Gutierrez, Ana Maria Ortiz, Gabriel Herrero-Beaumont, Jose L Pablos, Javier Narvaez, Federico Navarro, Jose L Marenco, Juan J Gomez-Reino, Antonio Gonzalez

**Affiliations:** 1 Laboratorio de Investigacion 2 and Rheumatology Unit, Hospital Clinico Universitario de Santiago, Santiago de Compostela, 15706, Spain; 2 Rheumatology Unit, Hospital La Paz, Madrid, 28046, Spain; 3 Laboratorio de Investigación Osteoarticular y del Envejecimiento, Servicio de Reumatología, Hospital Universitario Juan Canalejo, A Coruña, 15006, Spain; 4 Arthritis Unit, Rheumatology Department, Hospital Clinic, Institut d'Investigacions Biomèdiques August Pi I Sunyer, Barcelona, 08036, Spain; 5 Rheumatology Unit, Hospital Universitario Virgen de las Nieves, Granada, 18001, Spain; 6 Rheumatology Unit, Hospital Universitario Gregorio Marañon, Madrid, 28007, Spain; 7 Rheumatology Unit, Hopital Santa Creu e San Pau, Barcelona, 08025, Spain; 8 Rheumatology Unit, Hospital Clinico San Carlos, Madrid, 28040, Spain; 9 Rheumatology Unit, Hospital Universitario La Princesa, Madrid, 28006, Spain; 10 Rheumatology Unit, Fundación Jimenez Diaz, Madrid, 28040, Spain; 11 Rheumatology Unit, Hospital 12 de Octubre, Madrid, 28041, Spain; 12 Rheumatology Unit, Hospital Universitario de Bellvitge, Barcelona, 08907, Spain; 13 Rheumatology Unit, Hospital Univesitario Virgen Macarena, Sevilla, 41003, Spain; 14 Rheumatology Unit, Hospital Universitario de Valme, Sevilla, 41014, Spain; 15 Department of Medicine, University of Santiago de Compostela, Santiago de Compostela, 15705, Spain

## Abstract

**Introduction:**

Genome-wide association studies of rheumatoid arthritis (RA) have identified an association of the disease with a 6q23 region devoid of genes. *TNFAIP3*, an RA candidate gene, flanks this region, and polymorphisms in both the *TNFAIP3 *gene and the intergenic region are associated with systemic lupus erythematosus. We hypothesized that there is a similar association with RA, including polymorphisms in *TNFAIP3 *and the intergenic region.

**Methods:**

To test this hypothesis, we selected tag-single nucleotide polymorphisms (SNPs) in both loci. They were analyzed in 1,651 patients with RA and 1,619 control individuals of Spanish ancestry.

**Results:**

Weak evidence of association was found both in the 6q23 intergenic region and in the *TNFAIP3 *locus. The rs582757 SNP and a common haplotype in the *TNFAIP3 *locus exhibited association with RA. In the intergenic region, two SNPs were associated, namely rs609438 and rs13207033. The latter was only associated in patients with anti-citrullinated peptide antibodies. Overall, statistical association was best explained by the interdependent contribution of SNPs from the two loci *TNFAIP3 *and the 6q23 intergenic region.

**Conclusions:**

Our data are consistent with the hypothesis that several RA genetic factors exist in the 6q23 region, including polymorphisms in the *TNFAIP3 *gene, like that previously described for systemic lupus erythematosus.

## Introduction

The etiology of rheumatoid arthritis (RA) includes a genetic component that has become amenable to investigation in recent years. A major development has been the availability of large-scale genome-wide association (GWA) studies. The first GWA studies in RA were readily able to confirm the two clearest RA genetic factors – in the human leukocyte antigen region and in the *PTPN22 *gene [1-3]. In addition, such studies have found other significant associations. Some of these associations have already been confirmed in additional studies, such as the *TRAF1-C5 *locus and the intergenic region in the 6q23 chromosome [2-5].

**Figure 1:**
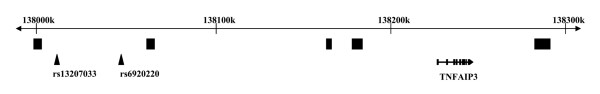
Map of the studied region in chromosome 6q23. Recombination hot-spots from the HapMap CEU data are represented as black squares below the rule showing physical distances along the chromosome in kilobases. The positions of the two rheumatoid arthritis-associated peak single nucleotide polymorphisms from previous studies (rs13207033 and rs6920220) are marked by arrowheads. The position of the *TNFAIP3 *gene and its structure are shown.

Two single nucleotide polymorphisms (SNPs) in 6q23, namely rs6920220 and rs13207033 (or its perfect surrogate rs10499194), have exhibited peak association with RA in an independent manner [2]. This finding has been interpreted as indicating the involvement of multiple genetic variants in RA susceptibility [2]. The associated region does not contain any known protein-coding sequence and lacks any evident functional consequence [2,4], but a strong RA candidate gene, the tumor necrosis factor-α-induced protein 3 (TNFAIP3) gene (also known as A20), is at about 185 kilobases (kb; Figure 1 shows the positions of these two loci). In addition, the rs6920220 SNP – together with SNPs in the *TNFAIP3 *gene – have been found to be reproducibly associated with systemic lupus erythematosus (SLE) susceptibility [6,7]. Therefore, we have hypothesized that genetic variation in *TNFAIP3 *could also be involved in susceptibility to RA. This gene is an excellent candidate for such an effect because it is a feedback negative regulator of tumor necrosis factor signaling through nuclear factor-κB (NF-κB) [8-10].

To test our hypothesis and to relate the *TNFAIP3 *locus to the intergenic 6q23 region, we have genotyped tagSNPs at both loci. Analysis in 1,651 RA patients and 1,619 control individuals revealed significant but weak associations at each locus. Each of these signals was statistically reinforced when signals in the other locus were accounted for. These results are consistent with multiple RA genetic factors in chromosome 6q23 that include polymorphisms in the *TNFAIP3 *gene and that interact with one another.

## Materials and methods

### DNA samples

Recruitment of samples included in this study has already been described [11]. Samples were obtained from Caucasian Spanish patients with RA (cases; n = 1,651) and control individuals (controls; n = 1,619). Cases and controls were recruited in different hospitals, and an attempt was made to match them by place of origin [see Table S1 in Additional data file 1]. All patients were classified in accordance with the 1987 American College of Rheumatology criteria [12]. Participants gave their informed consent and the ethical committees of participating centers approved the study.

### Single nucleotide polymorphism selection

Two separated regions of linkage disequilibrium (LD) in 6q23 were selected for analysis (Figure 1). The first, of 56 kb, includes the *TNFAIP3 *gene and the region of high LD with polymorphisms in the gene. The second, of 65 kb, is the LD region that includes the two association peaks from previous studies and that is limited by the recombination hot spots at about 138.002 and 138.067 megabases described in the HapMap CEU data (corresponding to samples with European ancestry) [13]. These two regions were 144 kb apart. TagSNPs [see Table S2 in Additional file 1] were selected from the HapMap CEU data using the Haploview software [14] to provide coverage with a pair-wise *r*^2 ^≥ 0.8 of all of the SNPs with minor allele frequency over 0.05 (*TNFAIP3 *locus) or 0.1 (intergenic locus). TagSNPs of the intergenic region included the peak SNPs in previous studies [1] and rs13207033, which is a perfect proxy of rs10499194 [2].

### Single nucleotide polymorphism genotyping

PCRs were done with the Qiagen Multiplex PCR kit (Qiagen, Valencia, CA, USA) on 30 ng genomic DNA. PCR products were purified by Exo-SAP digestion with Exonuclease I (Epicentre, Madison, WI, USA) and Shrimp Alkaline Phosphatase (GE Healthcare, Barcelona, Spain). Single-base extension reactions were done using the SNaPshot Multiplex Kit (Applied Biosystems, Foster City, CA, USA). Oligonucleotide sequences are presented in the additional materials [see Table S2 in Additional data file 1].

### Statistical analysis

Hardy-Weinberg equilibrium (HWE) concordance was tested in control samples. LD was analyzed using Haploview [14]. χ^2 ^tests for the 2 × 2 contingency tables were used to compare allele frequencies. The minor allele of each SNP was taken as reference for all comparisons, and minor allele frequencies are reported in the tables. Allele frequencies of each SNP were compared between controls from each center or region of origin, as a way to detect population heterogeneity. In addition, combination of results after stratification by individuals' origin was done following the Mantel-Haenszel approach, and heterogeneity of effect sizes was explored using the Breslow-Day test. ratio tests for the additive, dominant and recessive genetic models were obtained relative to the co-dominant model. Multivariate logistic regression analysis was used to evaluate the conditional effect of the SNPs. For conditioning on haplotype #5, a new genotype for this haplotype was created with codes 0 (non-carrier), 1 (heterozygote), and 2 (homozygote) for each individual. No problem of colinearity was detected with the inclusion in the model of haplotype #5 and SNP rs582757, which contributes to defining the haplotype, because the same allele of this SNP is present in three other common haplotypes. Stepwise logistic regression with all SNPs was conducted to detect the best multi-SNP models. Haplotypes were estimated using the Phase 2.1 software [15]. Odds ratios (ORs) for each haplotype were calculated taking as reference all chromosomes not bearing the haplotype. A customized version of Statistica 7.0 (Statsoft, Tulsa, OK, USA) was used for analyses except for statistical power, which was estimated using the 'Power and sample size calculations' software [16].

## Results

### *TNFAIP3 *locus

Six tagSNPs were sufficient to cover the *TNFAIP3 *gene and 20 kb of flanking sequences to either side [see Table S2 and Additional data file 1]. Genotypes of these tagSNPs were obtained with a 99.1% call rate, and they were in HWE except for the rs629953 SNP, which was excluded from further analysis.

No significant differences in allele frequencies were found between samples stratified by center of recruitment or region of origin. Analysis of allele frequencies revealed that the minor allele of the rs582757 SNP was significantly less frequent in patients with RA than in control individuals (OR = 0.89; Table 1). Genotype frequency comparisons yielded similar results (not shown). Estimation of the haplotype frequency distribution showed that only six haplotypes accounted for more than 98% of all chromosomes in patients with RA and controls (Table 2). The rs582757 alleles were distributed in several haplotypes and only the most common haplotype, which was defined by the major alleles of the five tagSNPs, was significantly different between patients with RA and control individuals (haplotype #5 in Table 2). Multivariate analysis combining haplotype #5 and rs582757 SNP genotypes revealed that any of them could account for the association with RA and that the two association signals were not independent (that is, association with the haplotype genotypes was not significant when conditioned on the rs582757 SNP, and *vice versa*) [see Table S3 in Additional data file 1]. This finding suggests that association is due to a causal polymorphism that is tagged by some haplotypes containing the T allele of rs582757 (Table 2). No significant change was detected by stratifying by sex (data not shown) or by the presence of rheumatoid factor (RF) or anti-citrullinated peptide antibodies (ACPAs) [see Table S4 in Additional data file 1].

**Table 1:** Comparison of allele frequencies in the tag-SNPs covering variability in the *TNFAIP3 *gene

SNP	MAF (% [*n*/*N*])	OR (95% CI)	*P*
rs600144			
Cases	25.9 (850/3278)	0.96 (0.9 to 1.1)	NS
Controls	26.8 (857/3202)		
rs11970361			
Cases	5.7 (187/3300)	0.94 (0.8 to 1.2)	NS
Controls	6.0 (194/3238)		
rs11970411			
Cases	10.2 (338/3300)	1.01 (0.9 to 1.2)	NS
Controls	10.1 (328/3238)		
rs582757			
Cases	24.8 (816/3286)	0.89 (0.8 to 0.99)	0.041
Controls	27.0 (871/3220)		
rs17780429			
Cases	12.7 (418/3300)	0.91 (0.8 to 1.0)	NS
Controls	13.8 (446/3236)		

Shown is a comparison of the allele frequencies in the tag-single nucleotide polymorphisms (SNPs) covering variability in the *TNFAIP3 *gene between patients with rheumatoid arthritis (cases) and control individuals (controls). MAF, minor allele frequency; *n*, number of minor alleles; *N*, total number of alleles.

**Table 2:** Distribution of estimated haplotype frequencies in the *TNFAIP3 *locus

Haplotype #	rs600144	rs11970361	rs11970411	rs582757	rs17780429	Controls (n [%])	Cases (n [%])	OR (95% CI)
1	T	C	G	**C**	**A**	427 (13.2%)	399 (12.1%)	0.92 (0.79 to 1.06)
2	T	**T**	**C**	T	G	185 (5.7%)	175 (5.3%)	0.93 (0.76 to 1.16)
3	T	C	G	**C**	G	443 (13.7%)	420 (12.7%)	0.93 (0.81 to 1.07)
4	**C**	C	G	T	G	849 (26.2%)	843 (25.5%)	0.98 (0.88 to 1.09)
5	T	C	G	T	G	1,159 (35.8%)	1,271 (38.5%)	1.14 (1.03 to 1.26)
6	T	C	**C**	T	G	141 (4.4%)	162 (4.9%)	1.15 (0.91 to 1.44)

Distribution of estimated haplotype frequencies in the *TNFAIP3 *locus for control individuals (controls) and patients with rheumatoid arthritis (cases). The minor allele of each tag-single nucleotide polymorphism is presented in bold. Haplotypes with frequency over 2% are shown, ordered by the odds ratio (OR) comparing cases with controls. Haplotype #5 was significantly different. CI, confidence interval.

### 6q23 intergenic locus

We have included 10 tagSNPs in addition to the two SNPs that have shown peak association in previous studies in the 6q23 intergenic locus [see Table S2 in Additional data file 1]. Genotypes of these 12 SNPs were in HWE and showed a high call rate (99.0%) in our samples.

Comparison of allele frequencies did not reveal clear association of any of these SNPs with RA (Table 3). Association in the peak rs13207033 SNP was completely absent. The rs6920220 SNP exhibited a trend (*P *= 0.07) in the same direction that has previously been reported [1,2,4]. Only the rs609438 SNP had a *P *value that was just below 0.05, with the minor allele exhibiting a lower frequency in patients with RA than in control individuals (Table 3). The difference was more marked when only women were considered (44.5% versus 48.9% in cases and controls, respectively; OR = 0.84, 95% confidence interval [CI] = 0.7 to 1.0; *P *= 0.006). Genotypes of this SNP were best fitted by a dominant model, and analysis according to this model revealed a clearer difference between patients with RA and control individuals (frequencies of CA + AA genotypes: 68.4% in cases versus 72.7% in controls; OR = 0.81, 95% CI = 0.70 to 0.95; *P *= 0.008). The difference was greater in women (frequencies of CA + AA genotypes in women: 66.8% in cases versus 74.0% in controls; OR = 0.71, 95% CI = 0.58 to 0.86; *P *= 0.0006).

**Table 3:** Allele frequencies of the 12 tagSNPs from the intergenic 6q23 region: cases versus controls

SNP	MAF% (*n*/*N*)	OR (95% CI)	*P*
rs566097			
Cases	11.4 (364/3,198)	0.99 (0.8 to 1.1)	NS
Controls	11.5 (367/3,182)		
rs13207033			
Cases	29.3 (897/3,062)	0.98 (0.9 to 1.1)	NS
Controls	29.7 (914/3,082)		
rs489738			
Cases	17.7 (559/3,164)	0.94 (0.8 to 1.1)	NS
Controls	18.6 (586/3,158)		
rs12194935			
Cases	23.0 (726/3,160)	1.00 (0.9 to 1.1)	NS
Controls	23.0 (726/3,154)		
rs536331			
Cases	42.1 (1,345/3,194)	0.99 (0.9 to 1.1)	NS
Controls	42.5 (1,351/3,182)		
rs675520			
Cases	48.6 (1,507/3,104)	0.96 (0.9 to 1.1)	NS
Controls	49.5 (1,520/3,070)		
rs6920220			
Cases	22.0 (703/3,200)	1.12 (1.0 to 1.3)	0.07
Controls	20.1 (639/3,178)		
rs694069			
Cases	39.0 (1,244/3,188)	0.96 (0.9 to 1.1)	NS
Controls	40.0 (1,273/3,180)		
rs6917441			
Cases	24.9 (790/3,178)	0.95 (0.9 to 1.1)	NS
Controls	25.8 (816/3,164)		
rs647108			
Cases	39.7 (1,265/3,188)	0.95 (0.9 to 1.0)	NS
Controls	40.9 (1,301/3,178)		
rs9321627			
Cases	22.7 (728/3,202)	0.96 (0.9 to 1.1)	NS
Controls	23.5 (749/3,186)		
rs609438			
Cases	45.1 (1,436/3,182)	0.91 (0.8 to 1.0)	0.047
Controls	47.6 (1,513/3,178)		

Shown are the allele frequencies of the 12 tag-single nucleotide polymorphisms (SNPs) from the intergenic 6q23 region in patients with rheumatoid arthritis (cases) and control individuals (controls). MAF, minor allele frequency; *n*, number of minor alleles; *N*, total number of alleles.

Genotype analyses of all of the other SNPs yielded findings similar to those of the allele frequency comparisons, and no differences were found by sex stratification in any of them (not shown). Two of the SNPs in this locus exhibited significant allele frequency differences between recruitment centers (rs6920220, *P *= 0.01; and rs675520, *P *= 0.004), and one of them was also different between regions of origin of the samples (rs6920220, *P *= 0.04). However, these differences did not introduce detectable artefacts in the global results, as shown by the similar results obtained above (Table 3) and with the Mantel-Haenszel approach (OR = 1.12 versus OR_M-H _= 1.12 for rs6920220; and OR = 0.96 versus OR_M-H _= 0.97 for rs675520) and lack of significant heterogeneity of the ORs as assessed with the Breslow-Day test (rs6920220, *P *= 0.5; and rs675520, *P *= 0.054).

Association of the rs6920220 SNP with RA has previously been reported to be stronger in patients with ACPAs or RF than in the ACPA-negative or RF-negative subgroup [4]. However, we did not detect any significant difference between these patient subgroups [see Table S4 in Additional data file 1]. In contrast, we found that the rs13207033 SNP was associated with RA only in the patients with ACPAs. Three other SNPs also exhibited significant association exclusively in ACPA-positive patients (Table 4). In all of these SNPs, the risk allele was the most common. Conditional logistic regression of these four SNPs, taken two by two, was unable to distinguish between them (data not shown). No association was found when the patient subgroups stratified by RF status were compared with control individuals (data not shown).

**Table 4:** Allele frequency differences in tagSNPs of the intergenic 6q23 region: ACPA-positive cases versus controls

SNP	MAF% (n/N)	OR (95%CI)	*P*
rs566097			
ACPA^+ ^cases	10.9 (84/772)	0.94 (0.7 to 1.2)	NS
Controls	11.5 (367/3,190)		
rs13207033			
ACPA^+ ^cases	25.1 (185/736)	0.80 (0.66 to 0.96)	0.015
Controls	29.7 (914/3,082)		
rs489738			
ACPA^+ ^cases	18.2 (138/760)	0.97 (0.8 to 1.2)	NS
Controls	18.6 (586/3,158)		
rs12194935			
ACPA^+ ^cases	26.1 (198/758)	1.18 (1.0 to 1.4)	NS
Controls	23.0 (726/3,154)		
rs536331			
ACPA^+ ^cases	37.9 (292/770)	0.83 (0.71 to 0.97)	0.023
Controls	42.4 (1,351/3,184)		
rs675520			
ACPA^+ ^cases	44.5 (334/750)	0.82 (0.70 to 0.96)	0.014
Controls	49.5 (1,520/3,070)		
rs6920220			
ACPA^+ ^cases	22.3 (172/770)	1.15 (0.95 to 1.39)	NS
Controls	20.1 (639/3,184)		
rs694069			
ACPA^+ ^cases	35.5 (273/770)	0.82 (0.70 to 0.97)	0.019
Controls	40.1 (1,276/3,186)		
rs6917441			
ACPA^+ ^cases	27.9 (212/760)	1.11 (0.9 to 1.3)	NS
Controls	25.8 (816/3,166)		
rs647108			
ACPA^+ ^cases	41.9 (321/766)	1.04 (0.9 to 1.2)	NS
Controls	41.0 (1,306/3,184)		

Allele frequency differences in tag-single nucleotide polymorphism (SNP) of the intergenic 6q23 region between patients with rheumatoid arthritis positive for anti-citrullinated peptide antibodies (ACPA^+ ^cases) and control individuals (controls). MAF, minor allele frequency; *n*, number of minor alleles; *N*, total number of alleles.

Haplotype analysis of the 12 tagSNPs in this locus did not reveal any significant association [see Table S5A in Additional data file 1]. Also, no significant difference was detected in haplotype frequencies between patients with ACPAs and control individuals [see Table S5B in Additional data file 1].

### Statistical interaction between the 6q23 intergenic locus and *TNFAIP3*

We found weak evidence of association with RA both in *TNFAIP3 *and in the 6q23 intergenic locus. SNPs from the two loci were not in LD (all values of *r*^2 ^< 0.07 in our samples). However, lack of pair-wise correlation does not exclude complex interdependence between the loci.

To explore more complex relationships, we used stepwise logistic regression with the 17 SNPs. This unsupervised multivariate process was run both in a forward and in a backward mode. That is, it was run starting with the most associated SNP and adding a SNP in each step until the model was not longer improved, or starting with a model incorporating all SNPs and eliminating the least associated in each step until the model deteriorated. The forward process yielded a best model that combined the rs6920220 SNP from the intergenic region and the rs582757 SNP from the *TNFAIP3 *locus (*P *= 0.009). The two SNPs were significantly associated when considered conditional upon the other (*P *= 0.027 and *P *= 0.013, respectively). The backward stepwise procedure yielded a best model with three SNPs (*P *= 0.009): two of the intergenic region, namely rs13207033 and rs609438, and the rs582757 SNP from the *TNFAIP3 *gene. Each made a significant contribution to RA when assessed conditional upon the other two (*P *= 0.046, *P *= 0.019, and *P *= 0.007, respectively).

Therefore, the two procedures showed that the best models differentiating cases and controls include SNPs from the two loci. In addition, they suggested interactions between them because the *P *values of association for each SNP were lower (more significant) in the multivariate analysis than when taken individually. For example, the rs6920220 and the rs13207033 SNPs were not associated with RA in isolation (*P *= 0.07 and *P *= 0.8, respectively), but they were associated in the multivariate models. If these results are confirmed, then they amount to epistasis between the two loci.

## Discussion

Association of RA susceptibility to SNPs in the intergenic region of chromosome 6q23 has attracted strong interest in a locus that, because of its lack of coding sequences, is especially difficult to investigate [1,2,4]. This was our motivation to study the clearest candidate among the genes flanking this locus, namely *TNFAIP3*. The recently reported coincident association in SLE increased our interest and suggested that the genetics of the region could be complex and include the *TNFAIP3 *gene [6,7].

Our findings regarding the *TNFAIP3 *locus revealed weak association with RA that could be explained either by the rs582757 tagSNP or by the commonest haplotype. Because of the weakness of the association and the multiple SNPs tested, these findings should be considered tentative. However, confidence in this association is increased by considering the summary statistics from the Wellcome Trust Consortium Case Controls (WTCCC) GWA study [1], which included 1,860 patients with RA and 2,938 healthy control individuals. That study yielded very similar results at the rs582757 SNP (25.0% versus 27.1% in RA patients and control individuals, respectively; OR = 0.90; *P *= 0.02). Additional preliminary data from a larger study are also concordant with association with RA of SNPs in the *TNFAIP3 *gene [17]. It is also of interest that a *TNFAIP3 *SNP with strong correlation (*r*^2 ^> 0.9) with the rs582757 SNP is associated with reduced expression of *TNFAIP3 *and with coronary artery disease in patients with type 2 diabetes [18].

Regarding the 6q23 intergenic region, we found weak association with a previously unreported SNP, rs609438, and replication of association with the rs13207033 SNP. This latter was observed only in patients positive for ACPAs. Previous reports could be interpreted as supporting this preferential association of the rs13207033 SNP, because association with this SNP was much clearer in the study conducted by Plenge and coworkers [2] (*P *= 4 × 10^-7^), in which all patients were ACPA positive than in the WTCCC GWA study [1] (*P *= 0.01), in which the patients were unselected. There are already antecedents of this type of preferential association in relation to the ACPA status of patients with RA, including the shared epitope, the *PTPN22 *nonsynonymous SNP and *IRF5 *[19-21]. However, specific analysis in other sample collections will be required to confirm this specificity of the rs13207033 association.

An unexpected result was the lack of association with RA of the rs6920220 SNP Association of this SNP with RA has previously been demonstrated in several studies with an overall OR of 1.23 [1,2,4]. Our study had enough power to detect this effect (power = 0.8 for *P *= 0.01). However, we found a weaker effect (OR = 1.12) than in previous studies. Such a difference in effect size between studies is common, and recent examples have been found in confirmed RA genetic factors such as *STAT4 *and *TRAF1-C5 *[5]. In these examples, and in many others, the observed effect sizes were weaker in replication studies than in the discovery study. This phenomenon has been characterized as the 'winner's curse'. It means that findings of discovery studies often overestimate the true association because they are conditional on those studies being the first to detect the association [22]. An alternative explanation for the lack of association in our study could be related to the differences in rs6920220 allele frequencies that were observed between recruitment centers. However, we consider this to be unlikely because the global comparison between patients with RA and control individuals showed that they were identical when the samples were taken as a whole or when they were stratified by place of origin.

The two large and comprehensive SLE studies of the 6q23 region have yielded multiple independent association signals, including polymorphisms in the *TNFAIP3 *gene [6,7]. At least three independent signals were detected in each of the two studies, although they were not completely concordant between each other or with the peak associations previously reported in RA. Our multivariate logistic analyses produced similar results, suggesting that polymorphisms in the two loci contribute to RA susceptibility. It therefore seems possible that this region contains multiple genetic factors shared by RA and SLE. Our data do not allow us to be more conclusive.

*TNFAIP3 *is a clear candidate for a role in RA by virtue of the anti-inflammatory effects of its encoded protein. It is involved in many regulatory feedback loops through the cooperative activity of its two ubiquitin-editing domains [9]. TNFAIP3 protein levels are drastically increased upon NF-κB stimulation by various factors, including tumor necrosis factor and interleukin-1 [9,10]. Once upregulated, TNFAIP3 inhibits NF-κB activity at multiple levels. Therefore, it seems likely that polymorphisms that reduce expression or function of TNFAIP3 will favor exaggerated inflammatory responses that may contribute to RA development and expression. However, our study has only provided suggestive evidence of the involvement of this gene in RA susceptibility. This evidence is reinforced by data from the WTCCC GWA study [1] and preliminary data presented at the 2008 American College of Rheumatology meeting [17]. Investigation of the 6q23 region should proceed with increased enthusiasm, given its likely involvement in multiple immune-mediated diseases and the possible involvement of *TNFAIP3 *– an important regulator of the NF-κB pathway.

## Conclusions

We have found evidence of multiple RA genetic factors in the 6q23 region including polymorphisms in the *TNFAIP3 *gene. These factors appear to be shared with SLE susceptibility. Involvement of TNFAIP3 is of practical interest, given its inhibitory effect on the NF-κB pathway. Nevertheless, there remain many aspects that require further analysis: confirmation of our results, delineation of genetic influences on specific RA subphenotypes, and identification of the functional variants in this locus and their effects.

## Abbreviations

ACPA: anti-citrullinated peptide antibody; CI: confidence interval; GWA: genome-wide association; HWE: Hardy-Weinberg equilibrium; kb: kilobases; LD: linkage disequilibrium; NF-κB: nuclear factor-κB; OR: odds ratio; PCR: polymerase chain reaction; RA: rheumatoid arthritis; RF: rheumatoid factor; SLE: systemic lupus erythematosus; SNP: single nucleotide polymorphism; TNFAIP3: tumor necrosis factor-α-induced protein 3; WTCCC: Wellcome Trust Consortium Case Controls.

## Competing interests

The authors declare that they have no competing interests.

## Authors' contributions

RD-G participated in design of the study, genotyped the samples, and participated in the interpretation of the results and in writing the manuscript. MC participated in the statistical analysis and in the interpretation of results. EPP, AB, FJB, JDC, RC, LC, ARS, BF-G, AMO, GH-B, JLP, JN, FN and JLM participated in the acquisition of clinical data and collection of samples, and in the analysis and interpretation of results. JJG-R coordinated the acquisition of clinical data and collection of samples, and participated in the analysis and interpretation of results. AG participated in the design of the study and in the coordination of acquisition of clinical data and collection of samples, and supervised genotyping, statistical analysis, interpretation of results and writing of the manuscript. All authors read and approved the final manuscript.

## Supplementary Material

Additional file 1 A Microsoft Word document that contains the following tables: Table S1 (distribution of samples by recruitment hospital), Table S2 (details of the SNPs that were studied and the oligonucleotides that were used), Table S3 (conditional analysis between the rs582757 SNP and the most common haplotype in the *TNFAIP3 *locus), Table S4 (results for each SNP stratified by ACPA or RF status), and Table S5 (haplotype analysis of the SNPs in the intergenic 6q23 region). Click here for file
