# Supplementary material for: Analysis of TNFAIP3, a feedback inhibitor of nuclear factor-κB and the neighbor intergenic 6q23 region in rheumatoid arthritis susceptibility
Source: Arthritis Res Ther. 2009 Mar 17;11(2):R42. doi: 10.1186/ar2650 (PMC2688189; doi:10.1186/ar2650)
Supplement: Additional file 1 — A Microsoft Word document that contains the following tables: Table S1 (distribution of samples by recruitment hospital), Table S2 (details of the SNPs that were studied and the oligonucleotides that were used), Table S3 (conditional analysis between the rs582757 SNP and the most common haplotype in the TNFAIP3 locus), Table S4 (results for each SNP stratified by ACPA or RF status), and Table S5 (haplotype analysis of the SNPs in the intergenic 6q23 region). [file ar2650-S1.doc]

| **Supplementary Table 1** |  |  |  |  |
| --- | --- | --- | --- | --- |
| Sample collections by hospital of recruitment | |  |  |  |
|  |  |  |  |  |
| **Center name** | **Town** | **Region** | **RA** | **Controls** |
| H Clínico Universitario de Santiago | Santiago | Galicia | 526 | 494 |
| Complejo H Univ Juan Canalejo | A Coruña | Galicia | 89 | 90 |
| H Clinic i Provincial | Barcelona | Cataluña | 47 | 47 |
| H de la Santa Creu I Sant Pau | Barcelona | Cataluña | 45 | 48 |
| H Príncipes de España | Barcelona | Cataluña | 93 | 92 |
| H Virgen de las Nieves | Granada | Andalucía | 92 | 94 |
| H Universitario de Valme | Sevilla | Andalucía | 71 | 94 |
| H Virgen de La Macarena | Sevilla | Andalucía | 164 | 140 |
| H Universitario de La Princesa | Madrid | Madrid | 107 | 98 |
| Fundación Jiménez Díaz | Madrid | Madrid | 34 | 45 |
| H Universitario 12 de Octubre | Madrid | Madrid | 85 | 74 |
| H Clínico San Carlos | Madrid | Madrid | 93 | 100 |
| H Universitario La Paz | Madrid | Madrid | 155 | 111 |
| H Universitario Gregorio Marañón | Madrid | Madrid | 50 | 92 |
| Total |  |  | 1651 | 1619 |

| **Supplementary table 2** | | |  |  | |  | |  |  |  |
| --- | --- | --- | --- | --- | --- | --- | --- | --- | --- | --- |
| List of the tagSNPs that were studied, including position, alleles and the used primers and probes. | | | | | | | |  |  |  |
|  |  |  |  | |  | |  |  |  |  |
|  |  |  | PCR primers | |  | |  |  |  |  |
| SNP ID | Position | Minor allele | forward | | reverse | | Minisequencing probe | Lenght of probe | Alleles in minisequencing | Alleles as in plus strand |
| rs566097 | 138002057 | T | tagaacagcagcaggcacat | | tggcaagaaaagacccaact | | CCTGGTGGTTGACTGgctgttacctcattgtgccactctagcctgaag Š | 48 | G>T | G>T |
| rs13207033 | 138007111 | A | tctattttatgctccatgggaaa | | ggtaatggactctctgaataccttg | | gaagtagaataattacttaaaagtccagtgttgtttaactcctctacagattt | 53 | C>T | G>A |
| rs489738 | 138023221 | C | agtcccttggaggttgtgac | | cctgcagcaatgcagattta | | tctggactttgagccccagagtaaaaatttaaccctct | 38 | A>G | T>C |
| rs12194935 | 138028843 | T | tgtcttggtcccttttgcat | | ccaaattgtgacttcaaccaa | | ccttgtattgaactaatatacagtttttaaaaataatttttaggggaaatgatgagatatgtc | 63 | C>A | G>T |
| rs536331 | 138034742 | C | aaagggaaatgcagagatgc | | acgacttgtatgcccctgat | | GCCTCCACGCACGTTGTGATAaaagggaaatgcagagatgcatttgatgtttttgggttgtga Š | 63 | T>C | T>C |
| rs675520 | 138034925 | G | gggcatacaagtcgtggatt | | gaaaatgtaaaagtaaccccaaaa | | atacaagtcgtggatttgactgggacaatttcttgtacatttccattgttttt | 53 | A>G | A>G |
| rs6920220 | 138048197 | A | tgctacggcagcgtaacata | | gaagcataaatttgcctcatca | | attgataaattatattttatctgcttccatctgttagcaggtaacttctccactaaaa | 58 | G>A | G>A |
| rs694069 | 138055244 | A | tcagcaacatcagcttccac | | tgtcccttccacgataggag | | ggatcagtgtcaatctctgttgctggta | 28 | C>T | G>A |
| rs6917441 | 138060132 | G | ggatggacttacccccaaat | | gtggagatggagccagtgat | | aagacaaggagccaagcttcacttatcaactcaacaca | 38 | T>C | A>G |
| rs647108 | 138064233 | G | ttatggggccagatgtcaag | | ggctgccctgtgtttatagg | | cagatgtcaaggacattttacaaaagagagccataccttaga | 42 | T>G | G>T |
| rs9321627 | 138064741 | A | agagcagaagcagggagttg | | cagatccactttgctcacca | | tgctctccctcatcagtgcatttaccat | 28 | G>A | G>A |
| rs609438 | 138064939 | A | tgagagcaagtgacgtgagg | | cctgcagcaatgcagattta | | CCTGGTGGTTGACTGtcagagcagctgccctgttagaacagggccaca Š | 48 | C>A | C>A |
| rs600144 | 138214076 | C | cctctttgggatgctcttca | | atgagtgtggctgtgttcca | | tttttttacatattcaatggttgaaaaaaattaaaaaatactttgtgccatatgaaaattata | 63 | T>C | T>C |
| rs11970361 | 138220829 | T | caaggctgttttggttggat | | gggtattttgcaagggagttc | | aggaactctaggattcctatgtagaaagtagccagaaaatgatcagtc | 48 | C>T | C>T |
| rs11970411 | 138220854 | C | caaggctgttttggttggat | | gggtattttgcaagggagttc | | gccagaaaatgatcagtcctgatggtttcgaagatctcaaact | 43 | G>C | G>C |
| rs629953 | 138236734 | A | ttgaaggaatactggctgtgg | | ttcaacccccaaacgataaa | | acaatttgataaaatttcaaatagctctattgccctacttatagagtaatgta | 53 | G>A | G>A |
| rs582757 | 138239517 | C | gcctggcctgttagctgtta | | tttgaatcctggctttgtca | | gtggaataagcactgtgctttggaaatattattttactgcatttttatccttttagca | 58 | T>C | T>C |
| rs17780429 | 138264281 | A | gaactgggacctgagatgga | | agtcaaccctgccgaatg | | ctgttttgccccagattggacctcttagatactgatac | 38 | C>T | G>A |
|  |  |  |  | |  | |  |  |  |  |
| Primers selected with Primer 3 and FastPCR softwares. They were checked to avoid formation of dimers between the primers included in the reaction | | | | | | | | |  |  |
| Š these oligonucleotides wete extended with a 5´ tail that has not homology with human sequences (these tails are in capiltal letters) | | | | | | | |  |  |  |

| **Supplementary table 3** | | |  |
| --- | --- | --- | --- |
| Conditional logistic regression analysis of association signals in the TNFAIP3 locus | | | |
|  |  |  |  |
|  |  | P values |  |
|  | unconditional | conditional on | |
|  |  | rs582757 | haplotype #5 |
| rs582757 | 0.042 |  | 0.231 |
| haplotype #5 | 0.016 | 0.062 |  |

| **Sup. table 4** | |  |  |  |  |  |  |  |
| --- | --- | --- | --- | --- | --- | --- | --- | --- |
| Allele frequencies of all the tagSNPs we have studied (in the intergenic region and in TNFAIP3) stratified | | | | | | | | |
| by ACPA and rheumatoid factor status | | |  |  |  |  |  |  |
|  |  |  |  |  |  |  |  |  |
|  |  | **ACPA status** |  |  |  | **RF status** |  |  |
|  | SNP | MAF% (n/N) | OR (95%CI) | p |  | MAF% (n/N) | OR (95%CI) | p |
|  | rs566097 |  |  |  |  |  |  |  |
|  | positive | 10.9 (84/772) | 0.94 (0.6-1.4) | ns |  | 11.5 /246/2146) | 0.90 (0.7-1.2) | ns |
|  | negative | 11.5 (42/364) |  |  |  | 12.6 (100/796) |  |  |
|  | rs13207033 |  |  |  |  |  |  |  |
|  | positive | 25.1 (185/736) | 0.79 (0.6-1.1) | ns |  | 28.5 (581/2038) | 0.95 (0.8-1.1) | ns |
|  | negative | 29.7 (104/350) |  |  |  | 29.6 (228/770) |  |  |
|  | rs489738 |  |  |  |  |  |  |  |
|  | positive | 18.2 (138/760) | 0.91 (0.7-1.2) | ns |  | 18.3 (389/2120) | 1.04 (0.8-1.3) | ns |
|  | negative | 19.7 (72/366) |  |  |  | 17.7 (139/784) |  |  |
|  | rs12194935 |  |  |  |  |  |  |  |
|  | positive | 26.1 (198/758) | 1.19 (0.9-1.6) | ns |  | 23.3 (493/2114) | 1.04 (0.9-1.3) | ns |
|  | negative | 22.9 (83/362) |  |  |  | 22.6 (178/786) |  |  |
|  | rs536331 |  |  |  |  |  |  |  |
|  | positive | 37.9 (292/770) | 0.81 (0.6-1.1) | ns |  | 41.0 (876/2146) | 0.88 (0.8-1.0) | ns |
|  | negative | 43.0 (160/372) |  |  |  | 44.0 (349/794) |  |  |
|  | rs675520 |  |  |  |  |  |  |  |
|  | positive | 44.5 (334/750) | 0.75 (0.6-1.0) | 0.026 |  | 48.0 (993/2070) | 0.94 (0.8-1.1) | ns |
|  | negative | 51.7 (183/354) |  |  |  | 49.6 (386/778) |  |  |
|  | rs6920220 |  |  |  |  |  |  |  |
|  | positive | 22.3 (172/770) | 1.01 (0.7-1.4) | ns |  | 21.7 (465/2144) | 0.94 (0.8-1.1) | ns |
|  | negative | 22.2 (83-374) |  |  |  | 22.7 (181/798) |  |  |
|  | rs694069 |  |  |  |  |  |  |  |
|  | positive | 35.5 (273/770) | 0.76 (0.6-1.0) | 0.033 |  | 38.6 (825/2138) | 1.00 (0.8-1.2) | ns |
|  | negative | 42.0 (153/364) |  |  |  | 38.7 (307/794) |  |  |
|  | rs6917441 |  |  |  |  |  |  |  |
|  | positive | 27.9 (212/760) | 1.38 (1.0-1.9) | 0.030 |  | 25.4 (539/2126) | 1.07 (0.9-1.3) | ns |
|  | negative | 21.9 (80/366) |  |  |  | 24.1 (191/792) |  |  |
|  | rs647108 |  |  |  |  |  |  |  |
|  | positive | 41.9 (321/766) | 1.17 (0.9-1.5) | ns |  | 40.1 (857/2136) | 0.99 (0.8-1.2) | ns |
|  | negative | 38.1 (141/370) |  |  |  | 40.3 (322/800) |  |  |
|  | rs9321627 |  |  |  |  |  |  |  |
|  | positive | 25.9 (200/772) | 1.29 (1.0-1.7) | ns |  | 23.3 (501/2152) | 1.11 (0.9-1.4) | ns |
|  | negative | 21.3 (80/376) |  |  |  | 21.4 (172/802) |  |  |
|  | rs609438 |  |  |  |  |  |  |  |
|  | positive | 48.6 (370/762) | 1.18 (0.9-1.5) | ns |  | 46.2 (986/2136) | 1.08 (0.9-1.3) | ns |
|  | negative | 44.5 (162/364) |  |  |  | 44.3 (349/788) |  |  |
|  | rs600144 |  |  |  |  |  |  |  |
|  | positive | 25.4 8196/772) | 0.99 (0.7-1.3) | ns |  | 26.3 8558/2120) | 1.05 (0.9-1.3) | ns |
|  | negative | 25.5 (96/376) |  |  |  | 25.4 (201/790) |  |  |
|  | rs11970361 |  |  |  |  |  |  |  |
|  | positive | 4.7 (36/772) | 0.92 (0.5-1.6) | ns |  | 5.5 (118/2134) | 1.05 (0.7-1.5) | ns |
|  | negative | 5.1 (19/376) |  |  |  | 5.3 842/794) |  |  |
|  | rs11970411 |  |  |  |  |  |  |  |
|  | positive | 9.6 (74/772) | 0.91 (0.6-1.4) | ns |  | 9.9 (211/2134) | 0.96 (0.7-1.3) | ns |
|  | negative | 10.4 (39/374) |  |  |  | 10.3 (82/796) |  |  |
|  | rs629953 |  |  |  |  |  |  |  |
|  | positive | 29.9 (230/770) | 1.05 (0.8-1.4) | ns |  | 28.3 (580/2052) | 1.03 (0.9-1.2) | ns |
|  | negative | 28.9 (108/374) |  |  |  | 27.6 (214/776) |  |  |
|  | rs582757 |  |  |  |  |  |  |  |
|  | positive | 26.7 (206/772) | 1.05 (0.8-1.4) | ns |  | 25.6 (544/2126) | 1.02 (0.8-1.2) | ns |
|  | negative | 25.8 (97/376) |  |  |  | 25.2 (199/790) |  |  |
|  | rs17780429 |  |  |  |  |  |  |  |
|  | positive | 12.8 (99/772) | 1.03 (0.7-1.5) | ns |  | 12.5 (267/2132) | 0.87 (0.7-1.1) | ns |
|  | negative | 12-5 (47/376) |  |  |  | 14.2 8113/796) |  |  |

| **Sup. table 5** | | |  |  |  |  |  |  |  |  |  |  |  |  |  |  |  |  |  |
| --- | --- | --- | --- | --- | --- | --- | --- | --- | --- | --- | --- | --- | --- | --- | --- | --- | --- | --- | --- |
| Haplotype frequency distribution of the 12 tagSNPs studied in the intergenic 6q23 region | | | | | | | | | |  |  |  |  |  |  |  |  |  |  |
| Haplotypes with frequency over 2% are shown, ordered by the OR comparing unseparated RA patients with controls | | | | | | | | | | | | |  |  |  |  |  |  |  |
|  |  |  |  |  |  |  |  |  |  |  |  |  |  |  |  |  |  |  |  |
| **A) Unseparated patients** | | |  |  |  |  |  |  |  |  |  |  |  |  |  |  |  |  |  |
|  |  |  |  |  |  |  |  |  |  |  |  |  |  | Controls |  |  | RA patients | | OR (95% C.I.) |
| haplo # | rs566097 | **rs13207033** | rs489738 | rs12194935 | rs536331 | rs675520 | **rs6920220** | rs694069 | rs6917441 | rs647108 | rs9321627 | rs609438 |  | n | % |  | n | % |  |
| 1 | G | G | **C** | G | T | **G** | G | **A** | A | T | G | A |  | 183 | 5.7 |  | 152 | 4.7 | 0.81 (0.65-1.02) |
| 2 | G | G | T | G | T | A | G | G | **G** | G | G | A |  | 85 | 2.7 |  | 72 | 2.2 | 0.84 (0.61-1.15) |
| 3 | **T** | G | **C** | G | **C** | **G** | G | G | A | T | G | A |  | 296 | 9.3 |  | 284 | 8.8 | 0.95 (0.80-1.12) |
| 4 | G | **A** | T | G | **C** | **G** | G | **A** | A | G | G | C |  | 876 | 27.5 |  | 866 | 26.9 | 0.97 (0.87-1.09) |
| 5 | G | G | T | G | T | A | G | **A** | A | G | G | C |  | 84 | 2.6 |  | 83 | 2.6 | 0.98 (0.72-1.33) |
| 6 | G | G | T | **T** | T | A | G | G | **G** | T | **A** | A |  | 626 | 19.6 |  | 626 | 19.5 | 0.99 (0.87-1.12) |
| 7 | G | G | T | G | T | A | **A** | G | A | G | G | C |  | 585 | 18.4 |  | 633 | 19.7 | 1.09 (0.96-1.24) |
|  |  |  |  |  |  |  |  |  |  |  |  |  |  |  |  |  |  |  |  |
|  |  |  |  |  |  |  |  |  |  |  |  |  |  |  |  |  |  |  |  |
| **B) ACPA+ patients** | | |  |  |  |  |  |  |  |  |  |  |  |  |  |  |  |  |  |
|  |  |  |  |  |  |  |  |  |  |  |  |  |  | Controls |  |  | RA patients | | OR (95% C.I.) |
| haplo # | rs566097 | **rs13207033** | rs489738 | rs12194935 | rs536331 | rs675520 | **rs6920220** | rs694069 | rs6917441 | rs647108 | rs9321627 | rs609438 |  | n | % |  | n | % |  |
| 4 | G | **A** | T | G | **C** | **G** | G | A | A | G | G | C |  | 876 | 27.5 |  | 213 | 24.5 | 0.87 (0.73-1.03) |
| 2 | G | G | T | G | T | A | G | G | **G** | G | G | A |  | 85 | 2.7 |  | 20 | 2.3 | 0.87 (0.53-1.42) |
| 3 | **T** | G | **C** | G | **C** | **G** | G | G | A | T | G | A |  | 296 | 9.3 |  | 75 | 8.7 | 0.93 (0.72-1.22) |
| 1 | G | G | **C** | G | T | **G** | G | A | A | T | G | A |  | 183 | 5.7 |  | 49 | 5.7 | 0.99 (0.72-1.37) |
| 5 | G | G | T | G | T | A | G | A | A | G | G | C |  | 84 | 2.6 |  | 20 | 2.3 | 0.88 (0.54-1.44) |
| 7 | G | G | T | G | T | A | **A** | G | A | G | G | C |  | 585 | 18.4 |  | 173 | 20.1 | 1.12 (0.93-1.35) |
| 6 | G | G | T | **T** | T | A | G | G | **G** | T | **A** | A |  | 626 | 19.6 |  | 192 | 22.3 | 1.18 (0.98-1.41) |
